# Factors associated with the recruitment of foreign nurses in Japan: a nationwide study of hospitals

**DOI:** 10.1186/s12960-020-00532-5

**Published:** 2020-11-10

**Authors:** Yuko O. Hirano, Kunio Tsubota, Shun Ohno

**Affiliations:** 1 Institute of Biomedical Sciences, Nagasaki University, 1-7-1 Sakamoto, Nagasaki, Nagasaki 852-8520 Japan; 2 The Nippon Agricultural Research Institute, 3-29. Kioi-cho, Chiyoda-ku, Tokyo, 102-0094 Japan; 3 Seisen University, 3-16-21, Higashigotanda, Shinagawa-ku, Tokyo, 141-8642 Japan

**Keywords:** Foreign nurse, Economic partnership agreement, Japan, Hospital

## Abstract

**Background:**

Nurse migration under bilateral agreements is a recent global trend, although lack of consultation with the health industries has led to challenges in the recruitment of foreign nurses by hospitals. To analyze the prevailing perception of hospitals on the Economic Partnership Agreement (EPA), under which Japan opened the doors to foreign nurses, we surveyed hospitals that are yet to employ foreign nurses.

**Methods:**

An anonymous questionnaire was developed and distributed to eligible hospitals; it assessed managers’ perception of Japan’s policy on the recruitment of foreign nurses and their intentions to hire foreign nurses under the EPA (hereafter called EPA nurses). We randomly selected 1879 hospitals, or 22% of the hospitals in Japan (*n* = 8540), with more than 20 beds. We used descriptive statistics, a Chi-square test, and logistic regression analysis to identify the predictors and developed a model to predict the likelihood of their intention to recruit EPA nurses in the future.

**Results:**

In total, 432 hospitals were eligible for further analysis (response rate: 22.9%). Half (50%) of the hospital managers were considerably interested in Japan’s policy on recruiting EPA nurses, although only 20% intended to recruit EPA nurses in the future. Willingness to recruit EPA nurses was associated with the degree of interest in the policy (OR 9.38; 95% CI 4.42–19.90) and managers’ perception of EPA nurses (OR 5.32, 95% CI 2.38–11.89).

**Conclusions:**

To attract more hospitals to recruit foreign nurses, it is essential for the Japanese government and the sending countries to review their EPA systems. Utilizing returning nurses to assist language acquisition by the forthcoming EPA nurses could be a provisional solution. For a more fundamental solution, long-term provision, from prior to their migration until their return migration, is needed to encourage brain circulation, as opposed to brain drain, between sending and receiving countries.

## Background

Nurse migration under bilateral agreements is one of the recent global trends, which can be observed in South Africa [1], ASEAN countries [2], Mexico [3], and India [4], and it may affect the migration flow of nurses by rapidly expanding market activities [5]. Previous studies on nurse migration under bilateral agreements indicated that bilateral agreements offer the flexibility to enable easier negotiation and quicker resolutions [6] between the signed countries. It aims to improve the relationship and accelerate the trade between source and target countries [7]. It also increases the temporary migration of nurses who offer healthcare services to countries facing significant shortages of nurses [8]. However, nurse migration under bilateral agreements adversely affects nurses. For example, it may lead to potential de-skilling [9] and render them vulnerable to the global economic crisis [3, 10]. Previous studies have indicated an insufficient involvement of health professionals in trade negotiations relating to public health [8, 11]; this may adversely impact nurses and healthcare institutions. Therefore, it is important to analyze the provision of bilateral agreements from the perspective of the healthcare industry.

In this study, we analyzed nurse migration under the Economic Partnership Agreement (EPA), signed between Japan and Indonesia (in 2007), between Japan and the Philippines (in 2006), and between Japan and Vietnam (in 2008). As of January 2019, only 136 foreign registered nurses remained in Japan [12], which is merely 10.5% of the 1300 EPA nurse candidates who had entered Japan since 2008.

**Fig. 1:**
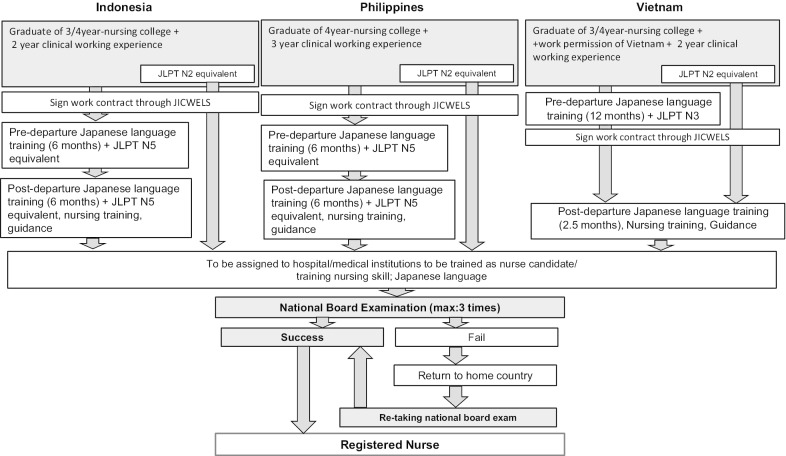
Flowchart of receiving EPA nurses in Japan by country

The flow of receiving EPA nurses is shown in Fig. 1. The qualifications and conditions for the eligibility criteria are stipulated in the EPAs signed between Japan and each of the source countries. Both applicants and the employer agree to the terms and conditions of training and work, and sign the contract. The employer pays a commission fee (131,400 yen per person) and a management fee (20,000 yen per person) to Japan International Corporation of Welfare Services (JICWELS), the only official agency for recruiting EPA nurses. Employers also pay 450 US dollars as agency fees to the Philippine Overseas Employment Administration of the Philippines for a Filipino nurse and the Department of Labor of Vietnam for a Vietnamese nurse. They pay 4055,000 Indonesian rupiahs (approximately 380,000 yen) to the National Board for the Placement and Protection of Indonesian Overseas Workers for an Indonesian nurse. In addition, Japanese language training fees, which costs 360,000 yen per person, are shouldered by employers [13]. Upon entry into Japan, EPA nurses are provided with a “Special Activities, Nurse Candidates” visa that is extensible for up to 3 years. After they pass the National Board Examination (NBE), they become entitled to the “Special Activities, Registered Nurse” visa, which can be extended indefinitely.

The focus of previous studies was that the system struggled to persuade EPA nurses to work as “nurse candidates” until they pass the NBE for nurses in the Japanese language to become “registered nurses” [14–17]. As “nurse candidates”, they cannot fully conduct nursing interventions, which they did in their country of origin. This system may disappoint EPA nurses. In fact, obtaining a high level of proficiency in the Japanese language is one of the biggest challenges for EPA nurses [15, 16, 18]. Differences in language, aside from differences in culture-based lifestyle, are considered barriers to the adjustment of Filipino [19], Chinese [20], Jordanian [21], and nurses of different nationalities who migrate to other countries [22, 23]. Further, the language skills have been shown to affect pass rates for those taking the English licensure exam [24, 25], and this may be worse when the exam is conducted in Japanese, as the language is less commonly spoken worldwide. EPA nurses also require additional effort to prepare for NBE. However, Japanese language proficiency does not necessarily guarantee success on the NBE [26] since the examination questions are based on Japan’s unique nursing practices [27]. For instance, Japan’s nursing practice involves “basic nursing tasks” including bed bath and toileting, and such nursing practices are particularly important in Japan, which is a super-aged society.

It is noteworthy that the challenges are faced not only by the nurses, but also by the hospitals employing EPA nurses. Especially at the outset of EPA, they struggled with instructing EPA nurses to pass the NBE, which is affected by the level of Japanese language proficiency of the EPA nurses [18]. The hospitals also struggled with differences in socio-cultural contexts between Japan and the EPA nurses’ countries of origin; such differences may widen the gaps in terms of nursing practice that diverges from the regulations or system of the country where these nurses originally worked [26]. This is a crucial detail when instructing EPA nurses to prepare for the NBE. This results in enormous costs to the hospitals, not only economically [28] but also psychologically [29], because they are responsible for assisting EPA nurses’ training and education [30].

The authors assume that Japan’s EPA, which underlies the “incompatibilities” between the right to employment and that to practice nursing [31], was not well consented by stakeholders [32, 33], especially at the outset of the EPA. Given sufficient information, the employment status of nurses would be well consented, and recruitment of EPA nurses would be better implemented. In this study, we analyzed the challenges of the EPA from the perspective of the hospital management. The study population included hospitals that had never accepted EPA nurses until the time of conducting the survey. This is due to the following reasons. First, some managers accepted EPA nurses after being asked by the Ministry of Health, Labour and Welfare (MHLW) to fill the EPA quota so they might receive benefits from the government that enable them to maintain a position of prestige in the medical community in Japan. They may be reluctant to disclose problems related to EPA nurses and may even disregard them because they wish to maintain links with the government [31]. Contrarily, hospitals that do not accept EPA nurses may express their perceptions more candidly, as they are not loyal to the government. Second, hospitals that do not accept EPA nurses may express their perceptions freely, which widens our view on recruiting EPA nurses. However, only a few studies have been conducted on this study population to investigate factors associated with the employment of EPA nurses in terms of hospital management. This study investigated the EPA policy by analyzing the perceptions of the hospital managers who have not yet employed EPA nurses. They need not surmise the intentions of the government; therefore, they can express their perceptions freely and objectively evaluate the advantages and disadvantages of the EPA without any constraints.

## Methods

This study aims to investigate the factors associated with the recruitment of EPA nurses among managers of hospitals in Japan who have not previously employed these nurses. A questionnaire was developed and distributed to the hospitals randomly selected from the stratified target population.

### Respondents

Respondents included managers in Japanese hospitals that had not employed EPA nurses. The lists of registered hospitals in each prefectural branch of MHLW were used for sampling. The selection criterion used the number of hospitals per prefecture. We selected hospitals with more than 20 beds to meet the condition of the JICWELS [34]. Questionnaires were mailed to 2000 selected hospitals. Of these, 121 questionnaires did not reach their destination, because of closure or removal. The rate of sampling, therefore, was 22% (1879 of 8540 hospitals registered in Japan).

### Instruments

We developed an 8-page questionnaire by examining previous similar surveys on hospitals that employed EPA nurses and unpublished interviews conducted by the research team. The questionnaire contained questions about the following: (1) the attributes of the hospitals, including their type (privately owned hospitals enacted by Medical Care Act Article 6 and publicly owned hospitals enacted by Medical Care Act Article 31) and the number of beds; (2) the possible factors that may affect the decision of recruiting EPA nurses (independent variables), including the difficulties in recruiting Japanese nurses, knowledge on recruiting EPA nurses, perceptions of EPA nurses, and perceptions of Japan’s policy on recruiting EPA nurses; and (3) the degree of willingness to recruit EPA nurses under the EPA program (dependent variable). The perception of EPA nurses was asked with respect to eight items on a four-point Likert-type scale (ranging from 1 = not agree at all to 4 = fully agree). The item scores were aggregated to an “image score” to represent the image of EPA nurses. The content validity was examined by the research team through discussions, and reliability was examined by Cronbach’s alpha. Respondents were divided into two groups by the average image score.

As for the policies on EPA nurses, respondents were asked about their views on the nine items listed in Table 2 and answered with a four-point Likert-type scale (ranging from 1 = do not agree at all to 4 = strongly agree). Willingness to recruit EPA nurses in the future under the EPA was measured on a four-point Likert-type scale (ranging from 1 = not at all to 4 = definitely will). Respondents were divided into two groups with average scores: those who were likely to recruit and those who were not.

### Data analysis

We used IBM SPSS Statistics for Windows, Version 25J for the statistical analyses. Descriptive statistics were used for the characteristics of hospitals and respondents, and Chi-square tests were used to test the correlation between the willingness to recruit EPA nurses, the attributes, and each independent variable. A logistic regression analysis was used to identify factors influencing willingness to recruit EPA nurses in the future. To develop the econometric model, eligible independent variables, selected from previous studies, were chosen from the list of perceptions on the government policy regarding the recruitment of EPA nurses, image score, and current conditions of recruiting Japanese nurses [18, 26, 28, 29, 35]. The independent variables were selected from the results of the Chi-square tests, which revealed statistical associations with dependent variables such as the intention to recruit EPA nurses in the future to be significant. A stepwise method was used to select the independent variables to develop the most appropriate models. The level of statistical significance was set at *p* < 0.05.

### Ethical considerations

Ethics approval was granted by the Biomedical Sciences Ethics Board at Nagasaki University (Permission number: 18030817).

## Results

### Respondents

A total of 485 hospital managers responded to questionnaires. Of these, 53 hospitals were excluded because they had either employed EPA nurses or did not answer the question. In total, 432 samples were analyzed (respondent rate: 23.0%) (Table 1).

**Table 1:** Characteristics of the respondents and willingness to recruit EPA nurses (*n* = 432)

Item	*n*	(%)	Willingness to recruit EPA nurses	*p* value
Respondents				
Directors/CEOs	166	38.4	22.6	0.651
Managers	163	38.2	18.9	
Head nurses	98	23.0	19.4	
Type of hospital				
Private hospitals	381	88.2	21.0	0.113
Public hospitals	50	11.6	12.5	
N.A	1	0.2		
Number of beds				
Less than 99	305	70.6	18.3	0.211
100–499	116	26.9	25.2	
500 and above	11	2.5	10.0	
Recruiting Japanese nurses				
Very hard/hard	347	80.3	21.8	0.047
Not so hard/not hard	75	17.4	12.5	
N.A	10	2.3		
Knowledge about EPA				
Know very much/much	136	31.5	19.4	0.473
Not so much/not at all	292	67.6	20.3	
N.A	4	0.9		
Interest in policies on EPA nurses				
Very much/much	216	50.0	34.9	< 0.0001
Not so much/not at all	211	48.8	4.8	
N.A	5	1.2		
Willingness to recruit EPA nurses in the future				
Very much/much	86	20.4	–	–
Not so much/not at all	336	79.6	–	–
N.A	10	2.3		

### Managers’ perception of EPA nurses

Approximately 96.3% of the hospital managers felt that training EPA nurses would be difficult, that EPA nurses found it difficult to master the Japanese language by working (94.8%), and that very few passed the NBE (93.4%). A large proportion also thought that accepting EPA nurses was costly (61.9%). However, a few managers thought that EPA nurses had high nursing skills (33.3%), acquired good reputations among patients and families (27.3%), remained in Japan for a long time (25.1%), and adjusted easily to Japanese hospitals (22.6%). Neither the type/size of hospitals nor the managers’ profession were significantly associated with the willingness to recruit EPA nurses. Therefore, in the following part of this study, we did not divide hospitals by type or size and did not view the manager’s profession as an influencing factor.

The mean image score was 15.17 (standard deviation [SD] = 2.98) with a minimum of eight and a maximum of 25. Cronbach’s alpha was 0.636. We divided the image score into two groups: lower image (less than 14) and higher image (15 or above).

### Perception of policy reform on recruitment of EPA nurses

The respondents’ perception of policy reform for EPA nurses is shown in Table 2 (left side).

**Table 2:** Perception of EPA policy reform by degree of intention to recruit EPA nurses (n = 432)

Perception of reform measures	Perceptions of the reform measures	Willingness to recruit EPA nurses in the future (n = 432)		
	Strongly agree/agree	Strongly agree/agree (%)	Not agree/not at all (%)	*p* value
Require nurses to pass certain level of Japanese language test before coming to Japan	85.5	19.1	80.9	0.067
Support hospitals in preparing nurses for the NBE	83.5	17.9	81.1	0.006
Require EPA nurses who pass the NBE to stay for several years	82.8	21.3	78.7	0.272
Organize to introduce EPA nurses to combat the shortage of nurses	70.8	26.1	73.9	< 0.0001
Invite private agencies under government supervision to stimulate the principle of market mechanism	58.1	22.6	77.4	0.115
Admit the multi-recognition of nursing licenses between Japan and the partner countries	56.8	26.5	73.5	< 0.0001
Admit *jun-kangoshi* (certified prefectural nurses) under the EPA	48.6	26.9	73.1	0.001
Prepare EPA nurses to pass the NBE	38.4	28.8	71.2	0.001
Not require EPA nurses to pass the NBE, but to accept them as Japanese nurses’ assistants	32.4	25.9	74.1	0.037

### Chi-square test

The results of the Chi-square test indicated that willingness to recruit EPA nurses was significantly correlated with perceived difficulties in recruiting Japanese nurses (*p* = 0.040), image score (*p* < 0.001), and degree of interest in Japan’s policy on EPA nurses (*p* < 0.001). The result of the Chi-square test between perception on policy and willingness to recruit EPA nurses in the future is shown in Table 2 (right side).

### Logistic regression analysis

Table 3 presents the final model of logistic regression, which was significant (*p* < 0.0001) and accounted for 37.6% of the variance (Nagelkerke *R*^2^ = 0.376).

**Table 3:** Factors affecting the willingness to recruit EPA nurses

Factors	OR	(95% CI)	*p* value
Interest in policies on EPA nurses	9.38	(4.42–19.90)	< 0.001
Image score	5.32	(2.38–11.89)	< 0.001
The Japanese government should articulate to introduce EPA nurses to overcome the shortage of nurses	3.09	(1.38–7.51)	0.013
The Japanese government should admit the mutual recognition of nursing licenses between Japan and the partner countries	1.89	(1.01–3.53)	0.048

## Discussion

Although the respondents had not employed EPA nurses previously, they offered several insights to identify the problems with and improve the policies on EPA nurses. The EPA nurse policies are not widely known, and hospitals in Japan are not adequately informed of them. Only 20% of the respondents answered that they wish to recruit EPA nurses. However, this does not necessarily mean that the rest are not interested in EPA nurses at all, as half (50%) of the respondents answered that they are interested in the government’s policy on EPA nurse recruitment. Given that over 80% of the respondents found it very hard/hard to recruit Japanese nurses, it may be interpreted that half of the respondents carefully monitor EPA policies, wondering if the current system would help fill the shortage of nurses. As such attitudes toward EPA nurses were observed regardless of type and size of the hospitals, or the managerial position of the respondents, and it seems that this perception is widely shared by hospitals in Japan.

One of the strongest factors associated with hesitation toward EPA nurses is their image. The image score mirrors a conviction of hospital staff that EPA nurses would encounter various difficulties arising from their limited Japanese language proficiency, which is crucial for their daily hospital work and for passing the NBE. We assume that there are some prejudices against EPA nurses. The respondents seem to be unaware of the many EPA nurses that had acquired a good reputation among patients and families. In fact, 75% of the hospitals that employed the first batch of Indonesian nurses in 2008 were satisfied or very satisfied with them because they had a bright personality (92.9%) and an appropriate attitude toward patients (89.3%) [35]. We assume that biases against EPA nurses are not the EPA nurses’ responsibility, but of the EPA system. The low Japanese language proficiency of EPA nurses may be due to an inadequate time period set for pre-departure Japanese language training. This is particularly true among nurses of the first, second, and third batches of nurses from Indonesia and the first and second batches from the Philippines, who were not given any pre-departure Japanese language training. It may be that the image scores of this study reflect the shortcomings of the EPA in the early stages. Vietnamese nurses, who have 12 months of pre-departure Japanese language training, have higher language proficiency. Therefore, they have fewer language problems than Indonesian or Filipino nurses. In this light, the governments of Japan and the sending countries are recommended to employ returning EPA nurses to teach the Japanese language and provide information on Japan’s unique nursing practices to the incoming EPA nurses, so that they will be well prepared to work and train in Japan. This may lessen the economic and psychological burden of hospitals.

This study also revealed the crucial conditions of hospitals that were struggling with a shortage of nurses. Twenty percent of the hospitals answered that they wished to recruit EPA nurses because they found it very hard/hard to recruit Japanese nurses. We assume that the hospitals attempted to employ EPA nurses to alleviate the shortage of Japanese nurses. A mid-term report released by MHLW [36] indicated that nursing shortages are especially severe in rural Japan. Hospitals in rural regions are assumed to be more likely to substitute EPA nurses for Japanese nurses than those in big cities, such as the Tokyo area. Regional differences cannot be proved by our survey because, due to the anonymity of the questionnaire, we could not identify the location of the hospitals. However, we take the excerpt from the narrative of a doctor respondent, who called the researchers to add comments on the survey, to strengthen the hypothesis. The respondent was the owner of a small private hospital but unfortunately was not successful in recruiting EPA nurses because nurses prefer to work in hospitals in the big cities. He expressed his opinion to the first author (HY) as follows: “I think the Japanese government should invite more EPA nurses to Japan. Please tell the government that there are some small-sized hospitals, especially in rural areas, that badly needs nurses. Even those who are from abroad will do to maintain our hospital to secure the health of people in this region.”

However, the current EPA system does not seem to be effective in solving the imminent problems of hospitals. The EPA sets a quota of 200 nurses per country per year, which falls well short of the deficit of nurses. The country needs at least 30,000 nurses by the year 2025 [37]. Nurses are needed in Japan, especially in rural areas, although EPA regulations seem not to secure them effectively. Therefore, a fundamental solution must be implemented.

Recently, hospitals in Japan interested in recruiting EPA nurses have noticed a higher number of Chinese nurses being hired through private agencies. The actual number of Chinese nurses is unclear, as the Japanese government does not disclose the nationality of persons who obtain “medical visas”, although the number of visas issued has been increasing. This phenomenon assumes that many Japanese hospitals are not satisfied with the system of recruiting nurses under the EPA and are thereby seeking other means of recruiting foreign nurses. Nearly 60% of the respondents of this study agreed to invite private agencies to the EPA under the supervision of the government to stimulate the principle of market mechanism, regardless of their intention of recruiting EPA nurses. The idea of implementing other means to address the manpower shortage seems to be widely accepted by hospitals nationwide. This is exemplified by the fact that the Japan Hospital Association, one of the biggest associations of hospitals in Japan, introduced the International Medical Human Resource Foundation in its website [38], a recruitment agency of Chinese nurses for member hospitals that claims to “provide a better service than the EPA”. According to the foundation [39], they recruit eligible Chinese nursing students to be educated in the Japanese language for 1 to 3 years prior to entering Japan. After entry, the Chinese nurses will enroll in Japanese language schools to improve their language skills to pass the N1 level (the ability to understand Japanese used in a variety of circumstances [40]) of the Japanese Language Proficiency Test to be eligible to take the NBE. After they pass the NBE, they can work in Japanese hospitals as registered nurses. Contrary to JICWELS, who clearly stated the recruitment costs and previous NBE passing rates of EPA nurses, these private agencies did not disclose such key information. Therefore, the authors are careful not to judge this business model prematurely, as many counties reported problems caused by improper nurse recruitment [41, 42]. Nonetheless, these recruitment agencies seem to be attracting more hospitals because Chinese nurses can read Chinese characters in the NBE, thereby presenting higher passing rates than EPA nurses. The new business model also attracts nurses. Since they are not entering Japan under the EPA, Chinese nurses are offered a “medical visa”. The visa is unlimited and allows nurses to work more flexibly than the “Special Activities, Registered Nurse” visa issued to EPA nurses. This flexibility is an advantage of the business.

Given these conditions, it is understandable that hospitals in Japan would be attracted to this model because, as shown in this study, 82.2% of the hospitals, regardless of their intention of recruiting EPA nurses, agreed with the statement “The Japanese government should require EPA nurses who pass the NBE to stay for several years.” This represents the severe need of the hospitals suffering from the shortfall of nurses. They wish for nurses to work for as long as possible. If nurses are given visas that permit more flexible work, they would spend more time working in Japan. If Japan wishes to employ more EPA nurses, attracting more hospitals by reviewing the EPA scheme so that it would satisfy the needs of the hospitals is key. As the logistic regression model indicates, the more hospitals that are interested in the EPA, the more chances they take to recruit EPA nurses.

However, the Japanese government does not seem keen to fill the nursing job vacancies with EPA nurses. We assume that the MHLW, who is in charge of monitoring the EPA nurses’ recruitment, cannot articulate the need to introduce EPA nurses to secure the nurse labor force, even though they know that there is a nursing labor shortage, as per their estimation [37].

Yeats et al. underscored that bilateral agreements offer governments more control and regulatory discretion [6]. As long as the government of Japan denies the recruitment of foreign labor in the name of immigration policy [43], it can only explain the significance of the present EPA as follows: “introducing EPA nurses is not to combat the shortage of nurses in Japan, but to respond to the request of the partner countries to accept the nurses” [30]. However, the statement is not helpful in assisting hospitals with nurse shortages, especially in rural regions [36]. Its ambiguity also confuses Japanese taxpayers, who paid more than 380,000,000 yen per year [44] in 2016 toward the costs of EPA nurses. It is, therefore, necessary for the Japanese government to make clear that the main purpose of bringing EPA nurses to Japan is to address the shortage of nurses.

### Limitations

This study is not immune to respondent bias. The majority of participants were from privately owned hospitals (88.2%) and small institutions with fewer than 99 beds (70.6%). Recruiting foreign nurses will help to secure the nurse supply and reduce the risk of building an “inefficient medical service system” that was set out in the mid-term report published by the National Committee for Social Security in 2008 [45]. The report called for changes in hospitals in Japan, the majority of which are small privately owned hospitals with too few medical staff. Further research, including publicly owned hospitals equipped with more beds, is therefore needed to confirm these results.

## Conclusion

This study revealed that the current EPA system is not the solution to counter the deficiency of nurses in Japan. This is because, as a form of trade agreement, the governments treat EPA nurses as a commodity [9]. In bartering Toyota cars with Filipino nurses and liquified petroleum gas with Indonesian nurses, they disregard the challenges of hospitals as well as those of EPA nurses. Similarly, Latin American nurses who migrated to Spain under the bilateral trade agreements realized that credential approvals were not transparent [10]. The trade agreement, as discussed above, sometimes confuses hospitals and nurses of the receiving countries, as it prioritizes accelerating the relationship with other countries in the global economy. Therefore, there may not be harmony in policy decisions that can achieve both health policy goals and economic trade goals [8]. Notably, EPA may also be harmful to the source countries because the health workforce strategies of their governments, focused on exports, may frustrate the efforts to ensure an adequate workforce of nurses [6, 17]. Better inclusion of trade agreement provisions into domestic health care policies is suggested. For instance, integrating the nurse education sector and labor market dynamics of both the sending and receiving countries can be a solution. The authors recommend that the Japanese government, in cooperation with nurse sending countries, develop the educational institutions in each sending country, to train nurses in fields in which Japan has good practices (i.e., gerontology nursing or psychiatry nursing). This may contribute to enhancing the opportunities of graduates of such institutions to remain in their country, which is becoming an aged society, or go to other destination countries, including Japan (through the EPA), with highly skilled nursing. Furthermore, facilitating returning migrants [46] should also be considered. Under such a long-term provision, this system may contribute to avoiding de-skilling and brain drain, but rather encourage brain circulation between sending and receiving countries. Simultaneously, the Japanese government is recommended to alleviate the regional shortage of nurses by subsidizing hospitals, particularly in rural areas, to raise the basic salary and compensations of nurses, regardless of their nationality, so that they can practice in more decent working conditions. Both international and domestic intervention are crucial for developing fundamental solutions to create a sustainable health workforce.

## Data Availability

Data will be released upon completion of the study and is available upon reasonable request from the first author.

## Abbreviations

EPA: Economic Partnership Agreement
JICWELS: Japan International Corporation of Welfare Services
NBE: National Board Examination
